# Harnessing the Power of Leptin: The Biochemical Link Connecting Obesity, Diabetes, and Cognitive Decline

**DOI:** 10.3389/fnagi.2022.861350

**Published:** 2022-04-22

**Authors:** Patricia Grasso

**Affiliations:** ^1^Department of Medicine, Albany Medical College, Albany, NY, United States; ^2^Department of Neuroscience and Experimental Therapeutics, Albany Medical College, Albany, NY, United States

**Keywords:** cognitive function, diabetes, exercise, leptin mimetics, obesity, synthetic peptides

## Abstract

In this review, the current understanding of leptin’s role in energy balance, glycemic regulation, and cognitive function is examined, and its involvement in maintaining the homeostatic “harmony” of these physiologies is explored. The effects of exercise on circulating leptin levels are summarized, and the results of clinical application of leptin to metabolic disease and neurologic dysfunction are reviewed. Finally, pre-clinical evidence is presented which suggests that synthetic peptide leptin mimetics may be useful in resolving not only the leptin resistance associated with common obesity and other elements of metabolic syndrome, but also the peripheral insulin resistance characterizing type 2 diabetes mellitus, and the central insulin resistance associated with certain neurologic deficits in humans.

## Introduction

**FIGURE 1:**
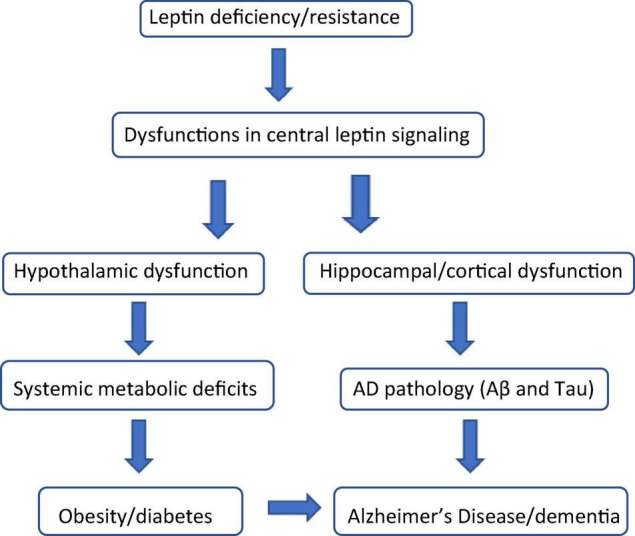
Leptin: the biochemical link connecting obesity, diabetes, and cognitive decline. Leptin deficiency (hypoleptinemia) and leptin resistance (hyperleptinemia) are characterized by dysfunctional leptin signaling both centrally and in the periphery. In the brain, dysfunctional leptin signaling in the hypothalamus causes numerous systemic metabolic defects including, but not limited to obesity and diabetes (insulin resistance). Dysfunctional leptin signaling in the hippocampus and cerebral cortex results in the induction and progression of the beta amyloid (Aβ) and Tau pathology associated with Alzheimer’s Disease (AD) and other forms of dementia. Obesity and diabetes are considered significant risk factors for the onset and progression of AD and AD-like cognitive impairment.

Since the cloning of the mouse and human leptin genes more than 25 years ago (Zhang et al., 1994), a significant body of research has been devoted to elucidating the biology and physiological role of leptin, the 16 kDa leptin gene product synthesized and secreted primarily by white adipose tissue (WAT). WAT, originally considered to be simply a fat storage depot, is now recognized as a highly-active endocrine organ which secretes a large group of hormones, collectively classified as adipokines or adipocytokines (Lehr et al., 2012). These hormones are pleiotropic in nature, and exert autocrine, paracrine, or endocrine influences on metabolic processes in the periphery and in the central nervous system (CNS) (Park and Ahima, 2015). Although leptin was originally considered an anti-obesity hormone because of its regulatory role in the maintenance of body weight (Friedman, 2016), there is growing evidence supporting the significant influence leptin exerts on both glycemic control (Lee et al., 2011; Morton and Schwartz, 2011) and cognitive function (Harvey, 2010; Yin et al., 2018; Forny-Germano et al., 2019) as well (Figure 1).

Sadly, by 2030 the number of overweight and obese adults is projected to rise to 1.35 billion and 573 million, respectively (Kelly et al., 2008). Without a doubt, lifestyle choices, nutrition, and the lack of sufficient exercise to control body weight have made obesity a well-established global risk factor for a number of other chronic disorders, including type 2 diabetes, cardiovascular disease, arthritis, mild cognitive impairment (MCI), Alzheimer’s Disease (AD), and some forms of cancer (Elias et al., 2005; Pugazhenthi et al., 2017; Lengyel et al., 2018). Although leptin therapy has been shown to reverse metabolic and neuroendocrine dysfunctions in obese individuals with congenital leptin deficiency, most cases of common obesity are associated with resistance to elevated levels of endogenous leptin, and to exogenously delivered leptin as well (Bluher and Mantzoros, 2009). This disappointing caveat has been a catalyst for the development of novel leptin mimetics which will be discussed later in this review.

In this review, the current understanding of leptin’s role in energy balance, glycemic regulation, and cognitive function will be examined; the effects of exercise on circulating leptin levels will be described; the notion that leptin may well be responsible for the homeostatic “harmony” of these physiologies will be explored; and evidence that synthetic peptide leptin mimetics may reduce or resolve the leptin resistance associated with these metabolic and neurologic deficits will be presented.

## Leptin and Obesity

### Background

Congenital leptin deficiency is rare, and is expressed in a phenotype which includes obesity, insulin resistance, diabetes, dyslipidemia, and other abnormalities associated with metabolic syndrome. When these patients are treated with leptin replacement therapy, appetite is suppressed, energy expenditure is stimulated, weight loss occurs, and insulin resistance, blood glucose levels, and dyslipidemia are reduced (Farooqi et al., 1999, 2000; Licinio et al., 2004). Common non-genetically obese patients, however, are refractory to exogenous leptin therapy and remain obese (Considine et al., 1996). The inability of leptin to exert an anorexigenic effect in these individuals reflects a state of leptin resistance in which extra calories are consumed and sustained weight loss is prevented (Woods et al., 2000). Paradoxically, although these individuals exhibit high levels of leptin expression in WAT, the elevated levels of circulating leptin fail to reduce adiposity, improve energy homeostasis, or reduce insulin resistance (Bluher and Mantzoros, 2009).

### Leptin Resistance

The mechanism(s) responsible for leptin resistance have been intensively investigated but are not yet completely understood. Over the years, a number of possible mechanisms have been suggested: downregulation of the leptin signal transduction pathway in hypothalamic and other CNS neurons known to regulate energy balance (Bjorbaek et al., 1998); impairment of leptin binding by elevated levels of C-reactive protein (Chen et al., 2006); decrease in histone deacetylase activity, an important regulator of food intake (Kabra et al., 2016); hypothalamic inflammation, endoplasmic reticulum stress, and autophagy (Mantzoros and Flier, 2000; Myers et al., 2012; Jung and Kim, 2013). There is no doubt that any or all of these abnormalities may contribute to the expression of leptin resistance and its associated dysfunctions.

Underlying all of these proposed mechanisms to explain the development of leptin resistance, however, are obesity-related defects in the saturable transport of leptin across the blood-brain barrier (BBB) into the CNS (Banks et al., 1999, 2004; El-Haschimi et al., 2000; Banks, 2015). In this regard, obesity and chronic consumption of high-fat diets are known to produce significant changes in the BBB (Kim et al., 2016), and also in brain regions containing neurons with high metabolic demands, such as those in the arcuate nucleus of the hypothalamus and the hippocampus (Moraes et al., 2009). Rodent studies have shown that maintenance on a high-fat diet not only compromises the integrity of the BBB due to the loss of leptin receptor-containing tanycytes, the specialized ependymal cells in the median eminence responsible for the active transport of leptin into the CNS (Balland et al., 2014; Kim et al., 2016), but also causes neuronal loss in the arcuate nucleus, the region of the hypothalamus containing first order neurons with the highest expression of leptin receptors (Moraes et al., 2009). Taken together, these observations suggest that reduced access of leptin into the CNS due to the depletion and/or desensitization of tanycytes (Banks et al., 2000), coupled with neuronal loss of leptin receptors may ultimately be responsible for the leptin resistance that characterizes common obesity.

### Mechanism of Action

Once across the BBB, leptin exerts its metabolic effects through binding to leptin receptors highly expressed in the arcuate nucleus of the hypothalamus and in some other brain regions (Moraes et al., 2009). Receptor binding initiates several signal transduction pathways: Janus kinase 2—signal transducer and activator of transcription 3 (JAK2-STAT3); insulin receptor substrate-phosphatidylinositol 3-kinase (PI3K); SH2-containing protein tyrosine phosphatase 2 (SHP2)-mitogen-activated protein kinase (MAPK); and 5’adenosine monophosphate-activated protein kinase (AMPK)/acetyl-CoA carboxylase (ACC) (Park and Ahima, 2015). Leptin activation of JAK2-STAT3 signaling, however, is the primary metabolic pathway regulating energy balance (Bates et al., 2003; Dardeno et al., 2010).

In the arcuate nucleus of the hypothalamus, leptin binding initiates a complex neural pathway which controls appetite by activating anorexigenic neurons that synthesize pro-opiomelanocortin (POMC) and cocaine- and amphetamine-regulated transcript (CART), hormones that inhibit orexigenic neurons that synthesize agouti-related peptide (AgRP) and neuropeptide Y (NPY), neurotransmitters that stimulate appetite (Ahima et al., 1999; Cowley et al., 2001; Robertson et al., 2008). Feeding is also suppressed by leptin binding to neurons in the lateral hypothalamus which express melanin-concentrating hormone (MCH) and orexins (Robertson et al., 2008). Also in the lateral hypothalamus, a distinct population of leptin receptor-bearing neurons which innervate the ventral tegmental area and do not express MCH or orexins, has also been identified suggesting an additional role for leptin in feeding behavior mediated by the mesolimbic dopamine system (Lenninger et al., 2009).

The role of leptin in energy balance has been most clearly demonstrated in rodent models of leptin deficiency. The ob/ob mouse, with a single point mutation at codon 105 in the leptin gene (Zhang et al., 1994), is totally leptin deficient, and presents with a phenotype including severe hyperphasia, a low basal metabolic rate, and rapid onset obesity (Pelleymounter et al., 1995), now known to result from over-expression of hypothalamic NPY and MCH, and low expression of POMC (Ahima and Osy, 2004). In this animal model, leptin treatment reverses all of these dysfunctions and normalizes synaptic inputs to POMC and AgRP neurons to levels found in wild-type non-obese mice (Pinto et al., 2004). As noted earlier, however, the majority of obesity in humans is not the result of leptin deficiency, but rather of leptin resistance—a critical barrier to leptin therapy.

## Leptin and Diabetes

### Background

The results of studies in genetically obese ob/ob mice (Koch et al., 2010), diet-induced obese (DIO) mice (Pocai et al., 2005), and insulin-deficient rats (Hidaka et al., 2002) indicate that intra-cerebroventricular (ICV) delivery of leptin, resulting only in insignificant changes in peripheral leptin levels, restores glycemic control and insulin sensitivity. These observations strongly support the notion that central leptin signaling alone is sufficient for the glucoregulatory actions of leptin, and that leptin affects peripheral insulin sensitivity *via* CNS mechanisms that are independent of its effects on food intake and body weight (Coppari and Bjorbaek, 2012).

### Central Gluco-Regulatory Effects of Leptin

It has been shown that expression of the long isoform of the leptin receptor is higher in the CNS than it is in peripheral tissues (Leshan et al., 2006). This observation has prompted studies designed to identify the specific brain regions involved in leptin-mediated glycemic regulation. These protocols have utilized the injection of leptin into specific brain areas, as indicated earlier, and genetic deletion or restoration of leptin receptors in specific neuronal populations (Fujikawa et al., 2013; D’souza et al., 2017). Leptin receptors are expressed principally in GABAergic and glutamatergic neurons in several regions of the hypothalamus: the arcuate (ARC) nucleus, the ventromedial nucleus (VMH), the lateral hypothalamic area (LHA), and the dorsomedial hypothalamic (DMH) nucleus (Schwartz et al., 1996; Elmquist et al., 1997, 1998). The primary neuronal populations, however, that have been associated with leptin-mediated effects on glycemic control are in the ARC and VMH (Elmquist et al., 1998; Coppari et al., 2005; Moraes et al., 2009).

A number of rodent studies have shown that leptin suppresses hepatic glucose production *via* multiple mechanisms, including some involving POMC- and AgRP-expressing neurons in the ARC. In this regard, selective expression of leptin receptors in hypothalamic POMC neurons has been shown to prevent diabetes in leptin receptor-deficient db/db mice, independent of changes in food intake and body weight (Coppari et al., 2005; Huo et al., 2009; Fujikawa et al., 2010; Berglund et al., 2012). Another study showed that selective re-expression of leptin receptors in AgRP neurons also mediates leptin’s anti-diabetic actions in db/db mice by suppressing glucagon production (Gonçalves et al., 2014). Interestingly, the loss of leptin receptors in POMC neurons of hyperleptinemic ob/ob mice did not affect the glucose lowering actions of leptin (Gonçalves et al., 2014). In streptozotocin (STZ) -treated mice, however, loss of leptin receptors in POMC neurons only partially prevented leptin-mediated reversal of hyperglycemia (Fujikawa et al., 2013). Taken together, these observations suggest that leptin action exclusively on POMC neurons is sufficient, but more than likely, not solely involved in the central regulation of blood glucose levels.

The CNS utilizes sympathetic nervous system (SNS) and parasympathetic nervous system (PNS) pathways to modulate peripheral responses, and a number of rodent studies have demonstrated the involvement of these pathways in the glucoregulatory actions of leptin. In lean rodents: (1) ICV or intravenous (IV) delivery of leptin has been shown to increase sympathetic activity in muscle, brown adipose tissue (BAT), kidney, and adrenal glands (Dunbar et al., 1997; Haynes et al., 1997), and parasympathetic activity in the liver (Tanida et al., 2015); (2) hepatic vagotomy in insulin-resistant mice only modestly inhibits the ability of leptin to improve glucose tolerance (Li et al., 2011); and (3) glucose uptake into BAT following the microinjection of leptin into the VMH of lean rats is blocked by surgical sympathetic denervation (Haque et al., 1999; Minokishi et al., 1999). In STZ-treated mice, however, the glucose-lowering action of leptin was not affected by partial chemical sympathectomy, sub-diaphragmatic vagotomy, or by antagonizing or blocking β-adrenergic receptors (Denroche et al., 2016). Taken together, the results of these studies provide substantial evidence supporting the involvement of the SNS and PNS in leptin-mediated glucose homeostasis.

### Peripheral Gluco-Regulatory Effects of Leptin

Insulin and glucagon, products of the β- and α-cells of the pancreas, respectively, are the principal regulators of glucose homeostasis. In addition to lowering blood glucose levels in hyperglycemic insulin-resistant ob/ob mice, leptin has been shown to reduce circulating insulin levels as well (Seufert et al., 1999). Leptin inhibits insulin gene expression and glucose-stimulated insulin secretion to adapt circulating glucose levels to body fat stores (Seufert et al., 1999; Cases et al., 2001), and insulin stimulates leptin synthesis and secretion establishing an adipoinsular axis (Seufert, 2004). This hormonal feedback loop, involving leptin from adipose tissue and insulin from β-cells, is vital to maintaining energy balance. Notably, the insulin reducing effects of leptin are not secondary to glucose lowering in ob/ob mice, however, since a reduction in circulating insulin was also observed in normal lean mice following leptin treatment (Seufert, 2004). Leptin has also been shown to protect β-cells from lipotoxicity in various animal models (Lee et al., 2007, 2011).

There is conflicting evidence as to whether or not leptin acts directly on β-cells to suppress insulin synthesis and secretion. Some studies utilizing pancreatic islets or perfused pancreas preparations have shown that leptin can inhibit insulin secretion (Emilsson et al., 1997; Fehmann et al., 1997; Ishida et al., 1997); others have shown just the opposite (Tanizawa et al., 1997; Leclercq-Meyer and Malaisse, 1998). Various leptin receptor knockout mouse lines, generated to investigate the role of leptin on β-cell function *in vivo*, have also produced conflicting results: LepR fl/flRIP-cre and LepRfl/fl Psdx1-cre mice are characterized by hyperinsulinemia (Covey et al., 2006; Morioka et al., 2007), whereas LepRflfl Ins1-cre mice do not exhibit hyperinsulinemia (Soedling et al., 2015). Adding to the confusion, neither RT-qPCR analysis (Soedling et al., 2015) nor single cell transcriptome analysis (Benner et al., 2014) of mouse and human islet cell populations has been able to detect leptin receptor on β-cells, in spite of published evidence of leptin binding, leptin receptor transcript expression, and functional leptin receptor signaling (Kiefer et al., 1996; Emilsson et al., 1997; Seufert et al., 1999; Benner et al., 2014).

In summary, although the evidence for direct effects of leptin on pancreatic β-cells is inconsistent, the indirect effects of leptin on insulin suppression are unambiguous. As reviewed earlier in this section, a number of observations support this assessment. The reports of reduced circulating insulin levels following ICV or systemic delivery of leptin in both genetic and non-genetic rodent models of diabetes, as well as those showing the induction of hyperinsulinemia in at least some strains of leptin receptor knock-out mice, strongly suggest that the regulation of insulin is mediated through the CNS.

## Leptin and Cognitive Function

### Background

In addition to its role in the regulation of energy balance and glucose homeostasis, a growing body of evidence implicates leptin in the maintenance of cognition and memory as well. Because extensive data from epidemiological studies have consistently confirmed that obesity, diabetes, and metabolic syndrome increase the risk of developing cognitive impairment and dementia (Baker et al., 2011; McCrimmon et al., 2012; Lehtisalo et al., 2016; Espeland et al., 2017; Pal et al., 2018), it is not surprising that more than 10 years ago, leptin was proposed to be the biochemical “link” connecting all of these pathologies (Harvey, 2010). This notion has been extensively explored since then, and appears to have weathered the test of time (Hamilton and Harvey, 2021).

### Neurotrophic Actions of Leptin

To understand the basis for the role of leptin in cognition, it is necessary to review the known effects of leptin on brain anatomy and function. Leptin receptors have been identified in both neuronal and non-neuronal cells not only in the hypothalamus, but also in the cerebral cortex, dentate gyrus, and in CA1 and CA3 areas of the hippocampus, brain regions known to be involved in cognition and memory (Schwartz et al., 1996; Pan et al., 2012; Kim et al., 2014). Several lines of evidence indicate a key role for leptin in neuro-developmental processes (Ahima et al., 1999). In this regard, leptin has been shown to regulate the morphology and synaptic function of hippocampal neurons (Irving and Harvey, 2014, 2021; McGregor and Harvey, 2019), and also to be essential for hippocampal spine formation (Dhar et al., 2014; McGregor and Harvey, 2019). In elderly humans, plasma concentrations of leptin are positively correlated with gray matter volume, and inversely correlated with age-related cognitive decline (Narita et al., 2009). Worthy of special note, humans with congenital leptin deficiency have been found to have structural brain abnormalities and neurocognitive deficits that can be attenuated by exogenous leptin (Matochik et al., 2005; Paz-Filho et al., 2008).

Communication between excitatory synapses can be modulated by changes in the level of neuronal excitation (Vitureira and Goda, 2013; McGregor and Harvey, 2019). Persistent increases, called long-term potentiation (LTP), or decreases, called long-term depression (LTD), in synaptic activity are proposed to be the principal cellular events involved in learning and memory (Bliss and Collingridge, 1993; Collingridge et al., 2010). The primary, but not sole, excitatory neurotransmitter in the mammalian brain is glutamate (Meldrum, 2000) which activates N-methyl-D-aspartate (NMDA) receptors, and leads to a postsynaptic rise in intracellular calcium that is critical for the induction of LTP and LTD at hippocampal CA1 synapses (Collingridge et al., 1983; Harvey, 2007). In this regard, leptin has been shown to modify excitatory synaptic transmission at hippocampal CA1 synapses by enhancing LTP and decreasing LTD, thereby increasing the efficiency of excitatory synaptic transmission, and improving hippocampal-dependent learning and memory (Shanley et al., 2001; Oomura et al., 2006; Moult and Harvey, 2011; Irving and Harvey, 2014, 2021). Interestingly, Durakoglugil et al. (2005) have reported that under conditions of enhanced excitably, however, leptin can also induce a form of NMDA receptor-dependent LTD. Taken together, these data provide strong evidence supporting the involvement of leptin in hippocampal function.

### Neuroprotective Actions of Leptin

In addition to the neurotrophic actions of leptin, its neuroprotective actions have also been demonstrated both *in vitro* and *in vivo*. In cell models of glucose deprivation, and in animal models of transient cerebral ischemia, leptin treatment has been shown to protect neurons from ischemic damage (Zhang et al., 2007; Zhang and Chen, 2008). Also, excitotoxic lesions in mouse brains generated by the delivery of the glutamate analog ibotenate, were significantly reduced by co-administration with leptin (Dicou et al., 2001). In other studies, leptin has also been shown to exert a cryoprotective effect in two experimental models of Parkinson’s disease, one induced by 1-methyl-4 pyridinium, a mitochondrial neurotoxin, and the other by 6-hydroxydopamine (Lu et al., 2006; Weng et al., 2007).

Leptin has also been shown to have neuroprotective effects against the progression of Alzheimer’s Disease (AD) pathology. A number of studies have consistently indicated that leptin treatment can decrease beta amyloid (Aβ) levels by targeting Aβ production, clearance, degradation, and aggregation (Fewlass et al., 2004; Marwarha et al., 2010; Greco et al., 2011; Niedowicz et al., 2013; Marawarha et al., 2014; Yamamoto et al., 2014). *In vitro*, exogenous leptin has been shown to protect hippocampal and hypothalamic cell lines from the neurotoxic effects of oligomeric soluble Aβ by preventing superoxide production and mitochondrial dysfunction (Yamamoto et al., 2014), and by reversing Aβ-mediated decreases in insulin-like growth factor-1, a known neuroprotective and neurotropic cytokine (Marwarha et al., 2011). Leptin has also been reported to reduce tau phosphorylation in primary cultures of murine cortical neurons (Doherty et al., 2013), in cultured rat neurons, and in human cell lines (Greco et al., 2008, 2009).

*In vivo* studies in a number of animal models have provided data supporting the *in vitro* data. Chronic leptin treatment of transgenic mice overexpressing amyloid precursor protein (APP) was shown to decrease brain levels of Aβ and phosphorylated tau (Greco et al., 2010). In other studies, both exogenous leptin administration and leptin viral gene transfer resulted in an improvement in memory and behavior tasks in transgenic mouse models of AD (Greco et al., 2010; Perez-Gonzalez et al., 2014). In rats, chronic ICV delivery of leptin was shown to reduce Aβ1-42-mediated impairment in spatial memory and suppression of hippocampal LTP (Tong et al., 2015).

### Role of Leptin in Alzheimer’s Disease

Recent epidemiological and human studies also indicate that leptin may play an important role in AD. It is well-documented that age is an important factor in the development of neurodegenerative disease. In this regard, low plasma leptin levels in late-life have been consistently associated with an increased risk for cognitive decline and the development of AD (Holden et al., 2009; Zeki Al Hazzouri et al., 2013; McGregor et al., 2014; Littlejohns et al., 2015; Yin et al., 2018; Lloret et al., 2019). In a number of studies, plasma leptin levels have also been reported to be lower in individuals with MCI (mild cognitive impairment) or AD compared to normal controls, and negatively correlating with the degree of cognitive impairment and dementia (Baranowska-Bik et al., 2015; Teunissen et al., 2015). Other studies have reported conflicting findings (Gustafson et al., 2012; Oania and McEvoy, 2015). It has been suggested, however, that the discrepancies in these studies may reflect the influence of confounding factors such as sample size, exercise, diet, sex, and even misclassification of AD as the diagnosis, that may not have been taken into consideration.

On the other hand, there is also a growing body of evidence from clinical studies implicating a significant role for inflammatory mechanisms in the pathophysiological processes leading to cognitive impairment and dementia (Kemka et al., 2014; Magalhães et al., 2018), and that cytokines participate in cognitive processes by influencing neuronal plasticity, neurogenesis, and neuromodulation (Marin and Kipnis, 2013; Donzis and Tronson, 2014). It is important to note here that leptin has a dual role: as a hormone, it influences multiple endocrine functions in addition to its key role in energy balance, and as a cytokine, it promotes inflammatory responses (La Cava, 2017). In this regard, leptin has been shown to enhance the expression and production of both TNF-α and IL-6 peripherally and in the brain (Magalhães et al., 2018; Feinkohl et al., 2020). TNF-α is known to modulate neuronal function by reducing hippocampal synaptic plasticity (DaRe et al., 2020). IL-6 has been associated with enhanced disease progression and severity of symptoms in AD (Donzis and Tronson, 2014). The pro-inflammatory actions of leptin have negatively impacted its clinical application to chronic human disease.

In suggesting that leptin may be the biochemical “link” connecting obesity, diabetes, and cognitive impairment, it seems necessary to comment on the seeming paradox regarding the observation that both mid-life obesity and late-life weight loss are considered risk factors for developing AD (Emmerzaal et al., 2015). In this regard, obesity (increased body fat mass) results in the production of pathologically high levels of circulating leptin and central leptin resistance, while being underweight (decreased body fat mass) results in the production of low levels of circulating leptin. The consequences of obesity or underweight are leptin resistance or leptin deficiency, respectively, which in both cases reduces leptin signaling in the brain. Because of leptin’s neurotrophic and neuroprotective actions discussed earlier, the deleterious effects of diminished leptin signaling on AD pathology and cognitive dysfunction are progressively enhanced.

## Leptin and Exercise

### Background

Hyperleptinemia, the consequence of increased leptin secretion and reduced leptin clearance, is a characteristic of the obesity syndrome (Hulver and Houmard, 2003). Counter-intuitively, since it is well-established that leptin induces satiety, stimulates lipid metabolism, and enhances energy expenditure (Baile et al., 2000; Dalamaga et al., 2013; Moon et al., 2013), one would not expect hyperleptinemia to be part of the pathology of obesity, but rather a part of its resolution. For reasons previously discussed, however, we know that hyperleptinemia is responsible for the leptin resistance seen in common obesity, and for the failure of exogenous leptin to resolve this problem. Thus, dietary interventions and exercise protocols designed to reduce plasma leptin levels may improve not only metabolic disorders associated with hyperleptinemia, but cognitive function as well.

Plasma leptin concentrations are tightly coupled to white adipose tissue mass (Ronnemaa et al., 1997). Consequently, any reduction in adipose tissue mass that occurs during weight loss lowers plasma leptin concentrations (Eriksson et al., 1999). Plasma leptin levels are also modulated by energy balance: they are increased by feeding and decreased by fasting (Kolaczynski et al., 1996a,b), and exhibit a diurnal circadian rhythm with highest concentrations near midnight and lowest concentrations near mid-morning (Friedman and Halaas, 1998; Kanabrocki et al., 2001). The regulation of this rhythm is also complex in nature. It is hormonally influenced (Nishiyama et al., 2000), sex-dependent (Paolisso et al., 1998; Saad et al., 1998), and altered by meal timing (Schoeller et al., 1997) and dietary composition (Choa and Boozer, 2000; Herrmann et al., 2001).

Because of these confounding influences, the results from studies examining the effects of physical exercise on plasma leptin concentrations are difficult to evaluate. Nonetheless, because leptin responses and adaptations to exercise may have important health-related ramifications, it is imperative that they continue to be examined. In this regard, exercise is known to not only effectively reduce body fat mass (Eriksson et al., 1999), but also to influence reproductive function (Veniant and LeBel, 2003) and thyroid function (Papovic and Duntas, 2005), as well as concentrations of other hormones, including insulin (Pocai et al., 2005), cortisol (Pralong et al., 1998), catecholamines (Fritsche et al., 1998), and growth hormone (Carro et al., 1997). Several cytokines, such as tumor necrosis factor–α and interlekin-6, also alter leptin mRNA expression and circulating leptin levels (Janik et al., 1997; Mantzoros et al., 1997).

### Effects of Exercise on Circulating Leptin

The effects of exercise on circulating leptin have been investigated under a number of different protocols: after single bouts of exercise at maximal and submaximal intensity, and for short and long durations; after short- and long-term exercise training; and after resistance (weight) training (Ryan et al., 2000; Hulver and Houmard, 2003). Cross-sectional data indicate that plasma leptin concentrations are associated with fitness level, and are not independent of body fat mass (Gippini et al., 1999). In order for exercise to alter serum leptin levels, a threshold of energy deficit must be achieved (Hulver and Houmard, 2003). When leptin levels are closely matched to adiposity at steady state, negative energy balance results in a reduction in leptin level that is more rapid than the change in adiposity (Morrison, 2008). In this regard, preventing this fall is sufficient to attenuate many of the physiological events associated with negative energy balance, such as a decline in metabolism, decreases in bone mass, reductions in thyroid hormones, reduction in testosterone levels, and inability to concentrate.

#### Single Bouts of Exercise and Leptin Levels

Studies examining the effects of a single bout of exercise of short and long duration, and at varying intensity levels, on circulating leptin levels in humans have reported conflicting results. With regard to short-term (<60 min) studies, Elias et al. (2000) observed a decline in plasma leptin in males after graded treadmill exercise to exhaustion, and suggested that this may be associated with an elevated production in non-esterified fatty acids during exercise, which has been shown to be inversely correlated with leptin levels (Duclos et al., 1999). Weltman et al. (2000) reported that 30 min of exercise at, above, and below lactate threshold, an index of accelerated metabolism and exercise intensity, did not alter serum leptin concentrations in young males either during exercise or recovery. Perusse et al. (1997) reported no change in leptin levels in 51 untrained men and 46 untrained women following a 10- to12-min maximal exercise test on a cycle ergometer. Fisher et al. (2001), however, observed increases in circulating leptin during 41 min of cycling at 50% of cycling intensity, followed by a reduction to control values during a 2-h recovery period. Kraemer et al. (1999) also reported significant increases in leptin responses in a graded exercise test to exhaustion in young adolescent runners, males and females, over the course of a short track season. Taken together, however, more studies suggest that plasma leptin concentrations in healthy males and females increase or remain unchanged by short-duration exercise, regardless of exercise intensity.

Similar to what has been reported for most short-duration exercise studies, the results of long-duration (>60 min) exercise studies have also shown either no change or a decline in circulating leptin following exercise. Torjman et al. (1999) measured leptin concentrations following 60 min of treadmill exercise at 50% of VO2max in six healthy males. After correcting for hemoconcentration, no effect on serum leptin during a 4-h recovery period was found. Landt et al. (1997) reported an insignificant 8% reduction in fasting serum leptin concentrations in 12 men after 2 h of cycling exercise. Leal-Cerro et al. (1998), in a study controlled for circadian variations, reported only a small reduction in serum leptin levels in males following a 26-mile marathon. Karamouzis et al. (2002) evaluated leptin responses in men following a 25 km ocean swim and reported reduced serum leptin concentrations. Zaccaria et al. (2002) reported the effects on serum leptin concentrations of three competitive endurance races in 45 males who participated in either a half-marathon run, a ski-alpinism race, or an ultramarathon race. Only the two prolonged endurance exercises involving high energy expenditure, the ski-alpinism race and the ultramarathon race, induced a marked reduction in circulating leptin.

#### Training and Leptin Levels

There have also been a number of studies reporting the effects of short- and long-term training, on leptin concentrations. In summary, short-term (<12 weeks) training has been consistently reported to have no influence on leptin concentrations in males or females unless the training was associated with fat loss (Halle et al., 1999; Houmard et al., 2000; Fatouros et al., 2005; Unal et al., 2005a,b; Kraemer et al., 2011).

There are disparate findings with respect to long-term (more than 12 weeks) training studies, however, with some studies reporting no effect of long-term training on leptin concentrations other than that induced by fat loss (Perusse et al., 1997; Fedewa et al., 2018), and others reporting a further reduction in plasma leptin after accounting for fat loss (Pasman et al., 1999; Thong et al., 2000; Roseland et al., 2001). Some of these contributing factors are suggested to involve alterations in energy balance, and improvements in insulin sensitivity (Flores et al., 2006) and lipid metabolism (Goldberg and Elliot, 1987).

#### Resistance Training and Leptin Levels

Other studies examined the effects of resistance exercise (weight training) on plasma leptin concentrations. In one of these, Kanaley et al. (2001) observed a reduction in plasma leptin in T2DM patients 24 h following upper and lower body resistance exercise, whereas non-diabetic individuals of the same age and sex did not show any decline. In another study, Nindl et al. (2002) measured leptin levels overnight at 3-h intervals following 50 total sets of squats, bench presses, leg presses, and pull-down exercise, and found a 3-h delay before any observable reduction in plasma leptin was evident. Ryan et al. (2000) examined the effects of resistance training in obese postmenopausal females, with and without weight loss. Fat-free mass and resting metabolic rate were increased in both groups, but plasma leptin was reduced by 36% only in the group that lost weight. In a recent meta-analysis (Rostas et al., 2017) examining 3481 medical records, training intervention was found to decrease plasma leptin in middle-aged or elderly (45 years and older), overweight or obese (BMI over 25) males and females, even without weight loss. Taken together, these results suggest that resistance training successfully reduces hyperleptinemia, even in the absence of dieting or major weight loss.

## Small Molecule Synthetic Peptide Leptin Mimetics

### Background

With the cloning of the mouse and human leptin genes (Zhang et al., 1994), as well as ongoing discoveries of the pleiotropic nature of leptin’s actions, it is not surprising that expectations for the therapeutic and/or prophylactic application of leptin to a number of metabolic and neurologic pathologies in the clinic were high. As discussed earlier, although leptin showed excellent results in reversing many metabolic (Friedman, 2016) and cognitive (Harvey, 2010) dysfunctions in rodent models, and in leptin deficient humans (Farooqi et al., 1999), clinical trials with recombinant leptin in subjects with common obesity consistently reported disappointing outcomes (Heymsfield et al., 1999; Chan et al., 2005; Lo et al., 2005; Zelissen et al., 2005). These results reflected the consequences of absolute or relative resistance to exogenous leptin caused by decreased efficiency, or failure, of leptin transport across the BBB into the CNS (Banks et al., 1999, 2000; Banks, 2015). Because of the potential adverse effects leptin, including its tendency to stimulate angiogenesis (Skrypnik et al., 2021) and carcinogenesis (Lin and Hsiao, 2021), and to aggravate autoimmune disease (Procaccini et al., 2015), the application of leptin to chronic disease has been severely limited. FDA approval has been given, however, to metreleptin, an analog of leptin, for treating lipodystrophy (Araujo-Vilar and Santini, 2019) because of its proven efficacy in treating leptin deficiency disease compared to its lack of efficacy in treating common obesity with hyperleptinemia.

Over the course of time, the limited clinical success of recombinant leptin has generated a great deal of interest, and effort, in developing leptin-related synthetic peptide drug candidates with the ability to cross the BBB, and mimick the central and peripheral activities of leptin. These candidates are small molecules corresponding to discrete domains within the leptin molecule—the domain(s) of interest being the active epitope(s) within the intact molecule. These efforts utilize the methodologies of solid phase peptide synthesis to construct the peptides, and rodent models in which the bioactivity of the peptides is assessed.

### Translational Pathway of a Small Molecule Synthetic Peptide Leptin Mimetic

#### Searching for the Active Epitope(s) of Leptin

Successful cloning of the mouse and human leptin genes led to the identification of a single point mutation at codon 105 which changes the coding sequence for the amino acid arginine at this position to a premature stop codon (Zhang et al., 1994). The consequences of the truncated mRNA transcribed by this mutation were reflected in a leptin deficiency syndrome that included obesity, increased body fat disposition, hyperglycemia, hyperinsulinemia, hypothermia, reduced bone mass, and impaired reproductive and thyroid function in both male and female ob/ob mice (Wauters et al., 2000). With this landmark discovery, peptide chemists now had some direction regarding where to start their search for active epitopes within the leptin molecule: in domains in the C-terminus distal to amino acid residue 105.

For two years, our laboratory used this information to initially epitope map the region between amino acids 106 and 167 (Grasso et al., 1997), and then the entire sequence of secreted mouse leptin (Grasso et al., 1999). Synthetic peptides, each 15 amino acids long and overlapping at the C-terminus by five amino acids, were delivered by intraperitoneal injection to leptin-deficient female ob/ob mice. In both of these studies, leptin-like activity was localized to a domain between amino acid residues 106 and 140 (85 and 119 in secreted mouse leptin after cleavage of the 21 amino acid signal peptide). Of the three peptides comprising this region that reduced body weight gain and food intake, the most active of these peptides encompassed amino acids 116–130 (85–109), and was chosen for further development. Confirming our choice, leptin-like activity of 116–130 on reproductive (Gonzalez et al., 1999; Tena-Sempere et al., 2000) adrenal (Malendowicz et al., 2000a,b), and cognitive (Malekizadeh et al., 2017) function was also reported.

#### Optimizing the Size of the Mimetic

Since then, a number of analogs of 116–130 have been developed in our laboratory, and their pre-clinical applications have expanded to include not only obesity, but also exploration of their efficacy in treating diabetes, osteoporosis, dyslipidemia, and cognitive dysfunction. Initially, C-terminal truncation of 116–130 indicated that of the 15 amino acids in this peptide, only the first seven (116–122) were essential for its activity (Rozhavskaya-Arena et al., 2000). This analog was called “OB3.” To improve the potency of OB3, and to increase resistance to proteolysis, the L-Leucine residue at position four (identified by alanine substitution as critical for its activity) in OB3 was replaced by its d-isomer (Rozhavskaya-Arena et al., 2000). This analog, called “[D-Leu-4]-OB3,” was significantly more potent than OB3 improved glycemic control as well as body weight gain (Grasso et al., 2001), and was the first analog to retain full activity after being delivered by intranasal instillation (Novakovic et al., 2009) or by oral gavage (Lee et al., 2010; Novakovic et al., 2010) in Intravail^®^ (Aegis Therapeutics, San Diego, CA), a patented transmucosal absorption enhancing agent.

In addition to its activity as stand-alone therapy, oral delivery of [D-Leu-4]-OB3 in Intravail^®^, in combination with exenatide or pramlintide acetate, augmented their effects on body weight gain and glucose homeostasis in an insulin-resistant mouse model (Leinung and Grasso, 2012). In insulin-deficient mice, [D-Leu-4]-OB3 was as effective as metformin in preventing the body weight gain associated with insulin therapy, and on a molar basis, was as good or surpassed metformin as an insulin sensitizer (Novakovic et al., 2013).

#### Defining the Signal Transduction Pathway of the Mimetic

Having confirmed the efficacy of our synthetic peptide leptin mimetics in a number of mouse models of obesity and diabetes, we turned our attention to exploring the molecular basis of this activity. We focused our efforts on the signal transduction pathways known to be involved in leptin signaling. In a specific and concentration-dependent manner, [D-Leu-4]-OB3 was found to induce phosphorylation of ERK1/2, STAT3 Ser-727, STAT3 Tyr-705, and PI-3K p85 (Lin et al., 2014). These results indicate that, in signaling pathways similar to those identified for leptin activation of STAT3, our biologically active leptin-related synthetic peptide analogs also activate STAT3 through phosphorylation of serine and tyrosine residues by multiple signal transduction pathways.

#### Improving the Pharmacokinetics and Efficacy of the Mimetic

With the possibility of future drug development for human disease, the pharmacokinetic (PK) profiles of [D-Leu-4]-OB3, following intraperitoneal (ip), subcutaneous (sc), and intramuscular (im) injection in PBS, and intranasal and oral delivery in Intravail^®^ were examined (Lee et al., 2010). The results of this study indicated that oral delivery of [D-Leu-4]-OB3 in Intravail^®^ was capable of achieving relatively high serum levels of bioactive peptide compared to commonly used injection methods. These profiles also suggested that the observed efficacy of [D-Leu-4]-OB3 on energy balance, glycemic control, and bone turnover in mouse models of obesity and/or diabetes might be improved by enhancing its bioavailability: i.e., increasing its uptake, extending its half-life, and reducing plasma clearance.

In an effort to address these issues, myristic (tetradecanoic) acid, known to increase membrane solubility, was conjugated to the N-terminal of [D-Leu-4]-OB3 (Novakovic et al., 2014). Myristoylation is the approach that was used to develop detemir insulin (Levemir^®^, Novo Nordisk), an analog of human insulin with a half-life of 7–8 h, which is commonly used in the management of T2DM in the clinic. This new analog was named “MA-[D-Leu-4]-OB3.” The PK profiles of MA-[D-Leu-4]-OB3 following ip, sc, and im injection in PBS, and intranasal and oral delivery in Intravail^®^ were compared to those of [D-Leu-4]-OB3. At a dose 10-fold lower than that used for [D-Leu-4]-OB3, the uptake and half-life of MA-[D-Leu-4]-OB3 were significantly elevated, and its plasma clearance was significantly reduced. Worthy of special note, compared to oral delivery of [D-Leu-4]-OB3 in Intravail^®^, uptake of MA-[D-Leu-4]-OB3 following oral delivery in Intravail^®^ was approximately twofold higher, half-life increased from 20 min to 29 h, and plasma clearance was reduced fivefold.

#### Visualizing the Uptake of the Mimetic in the Brain

The notion that leptin exerts its effects on energy balance and glycemic control *via* signals from the CNS was initially raised by Camfield et al. (1995). This hypothesis was subsequently confirmed, and a central mechanism of action regulating leptin’s effects on feeding behavior is now generally accepted. In this regard, studies utilizing both autoradiographic and *in situ* hybridization techniques have localized leptin receptors in the leptomeninges and choroid plexus of the third ventricle in ob/ob, db/db, and normal C57BL/6J mice (Lynn et al., 1996), and in the arcuate, ventromedial, paraventricular, and ventral premammillary nuclei of the hypothalamus (Mercer et al., 1996). Because of the uniquely high concentration of leptin receptors identified in the arcuate nucleus, this area of the brain is now recognized as the central leptin signaling center (Ha et al., 2013).

Based on physiological and signaling data, and the small size of our bioactive peptides, we hypothesized that they may be able to circumvent the transport defects associated with the leptin resistance seen in common obesity, and in a manner similar to leptin, achieve their effects on energy balance and glucose homeostasis through the activation of central leptin receptors known to be concentrated in specific nuclei in the hypothalamus. Because “seeing is believing,” free-floating coronal brain sections were processed and imaged by immunofluorescence microscopy following oral delivery of [D-Leu-4]-OB3 or MA-[D-Leu-4]-OB3 in Intravail^®^ to normal Swiss Webster and C57BL/6J mice, and to leptin-deficient ob/ob and leptin-resistant diet-induced obese (DIO) mice. In all four strains of mice, immuno-reactivity was concentrated in the median eminence, at the base and along the inner wall of the third ventricle, and in the brain parenchyma at the level of the arcuate nucleus (Anderson et al., 2017). These results provided visual evidence that [D-Leu-4]-OB3 and MA-[D-Leu-4]-OB3 cross the BBB, further supporting a central mechanism of action for these peptides. Most noteworthy in this study, however, was the localization of immuno-reactivity in the hypothalamus of DIO mice, *via* a conduit that is closed to leptin in this rodent model, and in most cases of human obesity.

### Assessing the Effects of Leptin Mimetics on Cognitive Function

Because the links between obesity, diabetes, and cognitive impairment are strong, and the regulatory effects of our synthetic peptide leptin mimetics on energy balance and glucose homeostasis have been established in a number of mouse models of obesity and diabetes, it was of interest to examine whether their influence on cognitive function also mimicked that of leptin. In STZ-treated Swiss Webster mice, a model of insulin deficiency (Anderson et al., 2019), and in DIO mice, a model of insulin resistance (Hirschstein et al., 2019), oral delivery of MA-[D-Leu-4]-OB3 in Intravail^®^ not only reduced body weight gain and restored glycemic control, but also improved episodic memory.

A recently-published follow-up study indicated that the mechanism of action by which MA-[D-Leu-4]-OB3 improves cognitive function in these mouse models appears to be related to its ability to enhance insulin sensitivity peripherally and in the brain, and to reduce TNF-α-induced neurodegeneration (Hirschstein et al., 2020). Most recently, the prophylactic capacity of MA-[D-Leu-4]-OB3 to prevent or slow the progression of obesity, insulin resistance, and cognitive impairment in a mouse model of T2DM has been reported (Chua et al., 2021). The ability of MA-[D-Leu-4]-OB3 to improve serum lipid profiles in mouse models of T1DM and T2DM (Hirschstein et al., 2021), suggests an additional application targeted toward reducing the risk of adverse cardiovascular events in humans that are associated with both T1DM and T2DM.

## Concluding Remarks

There is no doubt that obesity, diabetes, Alzheimer’s Disease (AD), and AD-like cognitive impairments are modern-day pandemics that are predicted to become even more wide-spread as world populations age. That these pathologies are linked to each other is obvious in that dysregulation of one becomes a risk factor for the development, or progression, of pathology in the others. In this review, we have examined the current understanding of the role of leptin, an adipocytokine hormone, in each of these pathologies, and discussed the reasons behind the disappointing outcome of its application in the clinic. We have reviewed the effects of exercise on circulating leptin levels, and have presented an over-view of research in the development of leptin mimetics, small molecule synthetic peptides containing the active epitope of leptin. These peptides have been found to safely mimick the activity of leptin in mouse models of genetic and non-genetic obesity, diabetes, and cognitive impairment, thus overcoming the central and peripheral leptin resistance responsible for leptin’s failure in the clinic. These studies provide convincing evidence supporting the notion that leptin mimetics may have the ability to “harness” the power of leptin, and may be useful in preventing or slowing the progression of leptin-regulated dysfunctions in human disease without the undesirable, and potentially lethal, side effects of leptin.

## Author Contributions

PG wrote the manuscript and approved it for publication.

## Conflict of Interest

PG was an unpaid scientific advisor to DIODEM Therapeutics, Inc., Albany, NY, United States

## Publisher’s Note

All claims expressed in this article are solely those of the authors and do not necessarily represent those of their affiliated organizations, or those of the publisher, the editors and the reviewers. Any product that may be evaluated in this article, or claim that may be made by its manufacturer, is not guaranteed or endorsed by the publisher.
